# Reactive Perforating Collagenosis; An Uncontrolled Pruritus That Left You Scratching Your Head

**DOI:** 10.7759/cureus.9175

**Published:** 2020-07-14

**Authors:** Danielle Kochen, Raman J Sohal, Amitpal Nat

**Affiliations:** 1 Internal Medicine, State University of New York Upstate Medical University, Syracuse, USA

**Keywords:** rare skin disease, pruritus, collagen, reactive perforating collagenosis, clinical dermatology

## Abstract

Acquired perforating collagenosis is a rare disease of altered collagen formation that is extruded through the epidermis. It is most commonly seen in patients with microvascular disease including longstanding diabetes and chronic kidney disease (CKD). Due to the rarity of the disease, no large randomized clinical studies have been performed to determine the most efficacious method of treatment. Therefore, most of the knowledge available for treatment is secondary to the information collected through case reports, case series, and retrospective analyses.

In this report, we present the case of a 68-year-old male with history of stage IV CKD who presented with a severe skin rash that was present over his body, including the chest, arms, back, neck, and buttocks. It did not involve the mouth, legs, palms or soles of the feet. He did not have a significant history of diabetes and had been placed on steroids with the concern that this rash may have been secondary to a drug reaction, erythema multiforme, or bullous pemphigoid. Two skin biopsies were performed as the patient was not responding to systemic and topical steroid or oral antibiotic therapy. The final biopsy ultimately revealed a diagnosis of acquired perforating collagenosis.

This is unusual in our case because although our patient had advanced CKD, he was not on dialysis, and had no significant longstanding history of diabetes. Additionally, as the prevalence of CKD is increasing in the population, it becomes more pertinent for providers to be aware of dermatological conditions associated with advanced CKD. This case report seeks to raise awareness of this disease. Furthermore, as the initial skin biopsy was unrevealing, this case also emphasizes the importance of repeating a biopsy to reduce the chance of sampling error.

## Introduction

Perforating dermatoses are a group of conditions in which there is an eruption of dermal connective tissue through the epidermis. There are four known primary disorders which are characterized by the specific content being eliminated through the epidermis. These four disorders include: Kyrle disease (keratin), reactive perforation collagenosis (collagen), elastosis perforans serpiginosum (elastin), and perforating folliculitis (follicle) [1]. There are two distinct forms of reactive perforating collagenosis (RPC): inherited form, a very rare form that manifests itself in infancy or early childhood, and acquired form.

Secondary perforating dermatosis, also known as acquired perforating dermatosis, encompasses all four perforating dermatoses and is associated with systemic diseases, specifically diabetes mellitus and chronic renal failure. This was replicated in a review of 22 cases of RPCs by Sayle et al. to determine the underlying diseases associated with this condition [1]. This report showed that 72% of these patients had chronic kidney disease (CKD) and 50% of them had diabetes mellitus. Of the patients with diabetes, 90% of them had CKD acquired secondary to diabetic nephropathy [2].

Although the specific pathogenesis and etiology are unclear, it is proposed that lesions develop at the site of trauma and are exacerbated by scratching [3]. Skin biopsy is mandatory for diagnosis; however, there have been no large randomized clinical studies that examine the most efficacious method of treatment. Treatment includes management of the underlying disease and maintenance of pruritus. Typically combinations of topical karatolytics, corticosteroids, emollients, or retinoids, along with oral allopurinol or antibiotics, have been prescribed [4]. Doxycycline has been used because of its anti-inflammatory properties. Resolution of the acute illness often results in disappearance of the acute skin lesions but will leave scarring with or without hyperpigmentation. 

## Case presentation

A 68-year-old male with a past medical history of chronic obstructive pulmonary disease (COPD) with chronic hypoxic respiratory failure requiring 5 L/min by nasal cannula, CKD stage IV, coronary artery disease, heart failure with preserved ejection fraction (68%), hypertension, peripheral vascular disease, and untreated Crohn’s disease presented to the emergency department (ED) with worsening rash along his chest, abdomen, back, bilateral arms, and buttock.

The patient was originally seen at an outside hospital where biopsy was taken preliminary for drug reaction, urticarial phase of bullous pemphigoid, or erythema multiforme. The biopsy was sent for further consultation, and he was discharged on a tapering dose of prednisone. He presented to our ED with the complaints of minimal improvement in his lesions. 

**Figure 1:**
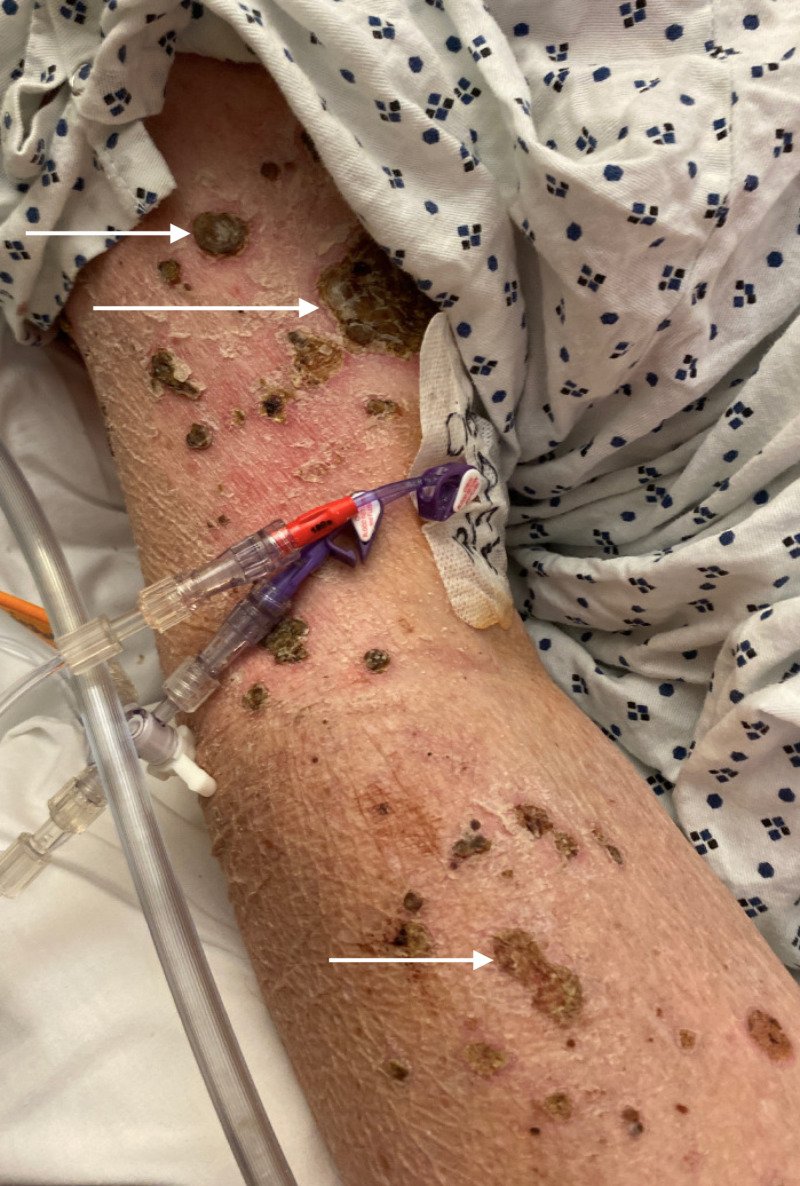
Right upper extremity skin lesions due to acquired perforating collagenosis

**Figure 2:**
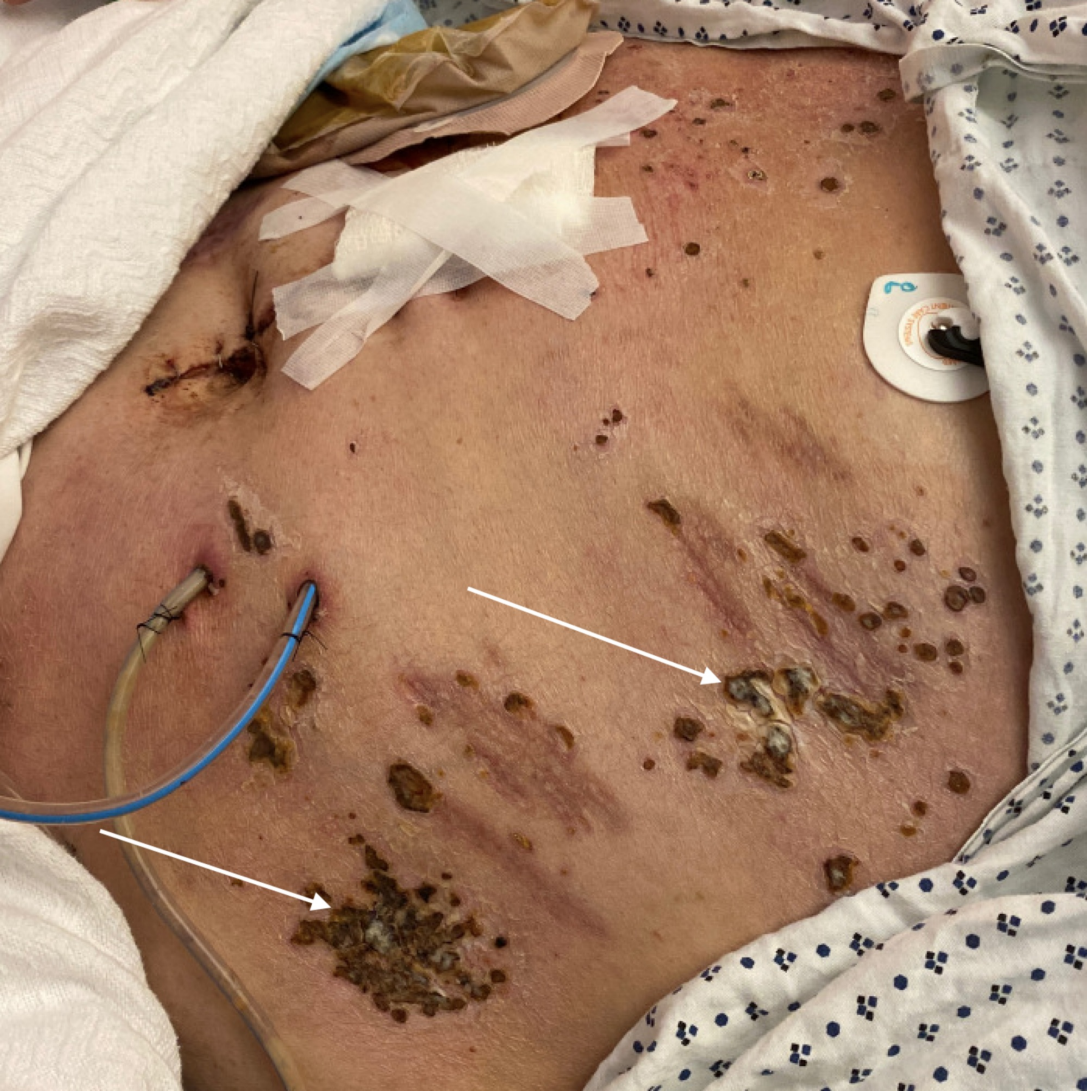
Abdominal skin lesions due to acquired reactive perforating collagenosis.

He was admitted to the outside hospital for COPD exacerbation with positive troponins and had a stress test preformed. He noticed the rash after receiving the technetium dye. He then began to develop erythematous, painful, burning, and pruritic lesions. His rash spared his palms, soles, and mouth. Due to his pruritus, the rash developed in to open wounds with crusting (Figures 1, 2). At our facility, laboratory investigations revealed negative results for antinuclear antibodies (ANA), cytomegalovirus (CMV), Epstein-Barr virus (EBV), and hepatitis B (Hep B) and hepatitis C (Hep C) titers. Additionally, Helicobacter pylori, fecal calprotectin, erythrocyte sedimentation rate (ESR), and C-reactive protein (CRP) were all within normal limits. Compliment studies, including CH50, C3, and C4, were also unremarkable. Dermatology was consulted, and a repeat skin biopsy from the abdomen and thigh was performed. Results showed broad epidermal ulceration with extensive scale crust and numerous vertically oriented collagen fibers perforating through the ulceration and scale crust suggestive of perforating collagenosis. Immunofluorescence showed no evidence of immunoglobulin or complement deposition in epidermis, dermal-epidermal junction, dermis, or blood vessels. The patient did mention that his mother and brother had the same lesions in the past, which had resolved on their own. 

On admission, he was started on solumedrol 60 mg IV every 12 hours and hydroxyzine for pruritus. He was then started on doxycycline 100 mg twice daily and dapsone. Ultimately on day 7, his solumedrol was changed to prednisone to begin a taper. For pruritus, first-generation antihistamines were used and an emollient was used for hydration. His pain was managed with morphine and gabapentin 100 mg three times daily. Triamcinolone 0.1% cream was additionally used. Doxycycline and dapsone were stopped once biopsy results returned. His lesions then began to scab, ultimately sloughing off resulting in residual scars. Additionally, the hospital course was complicated by perforated diverticulitis requiring exploratory laparotomy during which he had an abscess drained and a transversing loop colostomy created. There was a further recurrence of the abscess, along with wound dehiscence, for which he ultimately opted for comfort care measures. 

## Discussion

As mentioned above, our patient presented with a diffuse rash involving the abdomen, back, and upper extremities, while sparing the palms, soles, and mouth. Suspected etiologies at the time of admission included a hypersensitivity reaction, possible autoimmune etiology, or a vasculitic process. The preliminary skin biopsy suggested either the urticarial phase of bullous pemphigoid or erythema multiforme. Clinically, bullous pemphigoid can have a urticarial phase which would give a diffuse pruritic erythema with intermittent blister formation and crusted lesions, which were both consistent with our patient’s presentation. 

A second biopsy was done, this time sampling both affected and unaffected skin tissue of the abdomen and thigh. These biopsies were sent out for immunofluorescence. Due to the unrelenting pain and its minimal improvement on systemic steroid therapy, the patient was also started on dapsone and doxycycline. Both dapsone and doxycycline were selected because of their anti-inflammatory properties [1]. Although triamcinolone cream 0.1% was also used, a review of the literature shows that topical steroids have poor efficacy with unsatisfactory results for the treatment of RPC [4,5]. The intravenous solumedrol was changed to oral prednisone 70 mg twice daily on day 7 of admission, with a plan to taper by 5 mg every three days. Methotrexate was also considered as a possible option, given the patient’s history of untreated Crohn’s disease and the possibility of an autoimmune etiology. Other treatment options, including allopurinol, retinoids, and phototherapy, were not used to in our patient’s treatment. Allopurinol has been postulated to be effective as it can disruption the collagen glycation by inhibition of xanthine oxidase. Retinoids are postulated to be effective by stabilizing the keratinocytes [1]. Ultimately, the second biopsy report on day 8 suggested RPC without significant evidence of immunobullous dermatosis, vasculitis, lichenoid dermatitis, or hypersensitivity reaction. At this point, dapsone and doxycycline were discontinued. 

After an extensive workup and multiple biopsy reports, a final diagnosis of acquired perforating collagenosis was made. This case is interesting for several reasons. Firstly, this patient had a considerable family history of similar lesions, although they were not biopsy proven. Unfortunately, this information from the history was not strongly considered at the onset. Secondly, although this patient did have advanced CKD, he was not hemodialysis dependent as has been observed in many of the cases documented in the literature [6]. Other common comorbid disorders that are thought to be risk factors which were not present include hypothyroidism, hyperparathyroidism, dermatomyositis, or liver dysfunction [1]. Thirdly, he did not have a long-standing history of diabetes. The hyperglycemia was likely reactive due to high-dose steroids for several weeks. His hemoglobin A1c on admission was only 6.9 and likely reflected the recent changes in steroid dosing. 

Given the above atypical reasons, the time to diagnosis was prolonged and difficult to achieve. This case highlights the importance of repeating a skin biopsy, as the initial biopsy may be inaccurate due to sampling error. Furthermore, this is a rare disease and infrequently encountered by general physicians. It is found to typically occur in patients with extensive CKD and uncontrolled diabetes. With the growing prevalence of CKD in the general population the need to better understand the renal-associated dermatological conditions is essential. Therefore, this diagnosis should be considered in patients with diabetes, with further investigation especially prompted by strong family history.

## Conclusions

Acquired perforating collagenosis is a disease associated with significant morbidity. This is rare disease without clear guidelines for treatment. We hope that this case report will help shed light on this rare dermatological condition, which is associated with advanced CKD and with diabetes, with the hope that there is increased awareness of this condition raised, as the information that is currently available is through case reports and retrospective analysis.
